# Effect of dietary probiotics on the semen traits and antioxidative activity of male broiler breeders

**DOI:** 10.1038/s41598-018-24345-8

**Published:** 2018-04-12

**Authors:** Takio Inatomi, Konosuke Otomaru

**Affiliations:** 1 Inatomi Animal Clinic 4-18-25, Seijou, Setagaya, Tokyo, 157-0066 Japan; 2 Joint Faculty of Veterinary Medicine, Kagoshima University, 1-21-24 Korimoto, Kagoshima, 890-0065 Japan

## Abstract

This study aimed to investigate the effect of probiotics on the intestinal morphology, intestinal microflora, oxidative activity (biological antioxidant potential), and semen quality of male broiler breeders. For this, 180 Cobb male broiler breeders (60 weeks of age) were randomly distributed into two groups. The control group was fed a basal diet, and the probiotics group was fed basal diet supplemented with probiotics for 6 weeks. Probiotics containing *Bacillus amyloliquefaciens* TOA5001 improved the above mentioned characteristics of the male broiler breeders. Thus, *B. amyloliquefaciens* TOA5001 might improve the reproductive performance of male broiler breeders.

## Introduction

The sub-therapeutic use of antibiotics in animal nutrition has been found to be beneficial for the improvement of growth performance traits such as weight gain, feed efficiency, and mortality rate^1^. Antibiotics are used to improve the health and productive performance of animals used for human consumption; however, they also result in the emergence of drug-resistant microorganisms. The European Union has banned the use of antibiotics as growth-promoting agents in the poultry industry, and many countries are increasingly restricting the prophylactic use of antibiotics in animals raised for food. Therefore, the poultry industry is actively searching for alternatives to antibiotics, and many different functional preparations such as herbs, essential oils, organic acids, and probiotics have been tested.

Probiotics are living microorganisms that improve animal health when included in the diet. They act by balancing the intestinal flora, influencing intestinal villi, and improving nutrient digestion and absorption^2^. Supplementation of probiotics in the feed of chickens has been found to improve (i) growth and productive performances such as body weight, daily weight gain, carcass percentage, absolute organ weight and organ weight/body weight ratio^3–13^; (ii) nutrient digestibility^14–16^; (iii) intestinal microflora modulation^17–19^; (iv) pathogen growth inhibition^16^; (v) immunomodulation and gut mucosal immunity^11,16,18,19^; and (vi) antioxidant status^20^. Various microorganisms have been used as probiotics, including species of *Bacillus*, *Bifidobacterium*, *Enterococcus*, *Lactobacillus*, *Lactococcus*, *Streptococcus*, and numerous yeast strains. Although many studies have investigated the beneficial effects of probiotics in chickens, few have determined their effects on male broiler breeders. This study aimed to determine the effect of probiotics on the semen traits, intestinal morphology, intestinal microflora, and antioxidant status of male broiler breeders.

## Materials and Methods

### Ethical approval

This study was conducted at a commercial poultry farm in Kagoshima Prefecture, Japan, and performed under the fundamental guidelines for the proper conduct of animal experiments and related activities at academic research institutions under the jurisdiction of the Ministry of Education, Culture, Sports, Science and Technology. This study was approved by the Ethics Committee of the Inatomi Animal Clinic (Tokyo, Japan).

### Birds, diets, and management

In this study, 180 Cobb male broiler breeders that were 60 weeks old were used. The birds were randomly divided into 2 treatment groups (groups 1 and 2) of 90 birds each. Before starting this study, semen of all birds were analysed and there were no differences in semen trait in each group. The birds were individually placed for 6 weeks in cages having the following dimensions: 30 cm × 37.5 cm × 52.5 cm × 52.5 cm. Nipple drinkers and trough feeders were included in the cages. All birds were housed in a windowless and environmentally controlled room, with the room temperature maintained at 22 °C to 24 °C. The illumination period was 16 h·d^−1^. The birds in group 1 were fed a basal diet, and those in group 2 were fed basal diet containing probiotics for 6 weeks. The probiotics (TOA Pharmaceutical Co. Ltd., Tokyo, Japan) containing *Bacillus amyloliquefaciens* TOA5001 at 1 × 10^8^ colony-forming units·g^−1^ in rice bran was supplemented to the feed at 0.2% (w/w). The basal diet consisted of commercially available antibiotic-free male broiler breeder feed (Kagoshima Agricultural Economic Federation). The basal diet was used in a mashed form and formulated to meet the nutritional requirements of 60–66-week-old male broiler breeders as per the guidelines of the Kagoshima Agricultural Economic Federation. The composition of the basal diet and nutrient content are shown in Table 1. The diet for both the groups was provided at 125 g feed per bird per day, and water was provided *ad libitum*.

**Table 1:** Composition of non-antibiotic basal diets.

**Ingredient**	
Maize (%)	51.5
Soybean meal (%)	12.5
Sunflower extract (%)	5
De-oiled rice bran (%)	27.1
Mineral mixture¹ (%)	3
**Additive**	
Salt (g/100 kg)	400
A B_2_D_3_K² (g/100 kg)	20
B-complex³ (g/100 kg)	20
Choline chloride^4^ (g/100 kg)	100
d/l-Methionine (g/100 kg)	180
Lysine (g/100 kg)	170
**Calculated analysis**	
ME (MJ/kg)	10.7
CP (%)	15.6
Fibre (%)	7.55
Calcium (%)	1.04
Available phosphorus (%)	0.34
Lysine (%)	0.88
Methionine (%)	0.45

¹Contained: Ca, 32%; P, 6%; Mn, 0.44%; Zn, 0.33%; I, 150 ppm; Fe, 2000 ppm; Cu, 250 ppm; Se, 45 ppm.

²Contained per gram: A, 82,500 IU; D3, 12000 IU; B_2_, 50 mg; K, 10 mg.

³Contained per gram: B_1_, 4 mg; B_6_, 8 mg; B_12_, 40 μg; E, 20 mg; niacin, 60 mg; calcium pantothenate, 12.5 mg.

^4^Contained 60% choline chloride.

^5^Contained 78% l-lysine hydrochloride.

### Small intestinal morphology

After the semen, serum, and plasma samples were collected, all birds were killed at 66 weeks of age to collect intestinal segments. The intestinal segment samples were collected from the jejunum and ileum and, after their contents were flushed with physiological saline, the samples were submerged in a fixative solution (0.1 M collidine buffer, pH 7.3) containing 3% glutaraldehyde, 2% paraformaldehyde, and 1.5% acrolein. They were then brought to the laboratory to determine the morphological changes. Three cross-sections for each intestinal sample were prepared after staining with azure A and eosin by using standard paraffin embedding procedures^21^. A total of 10 intact, well-oriented crypt-villus units were selected in triplicate for each intestinal cross-section. The villus height, measured from the tip to the crypt junction, and crypt depth, defined as the depth of the invagination between adjacent villi, were measured using Image-Pro Plus (Media Cybernetics, Washington, USA) as described in detail by Touchette *et al*.^22^. The villus height:crypt depth ratio was also calculated.

### pH of digestive tract contents

The pH of the different parts of the gastrointestinal tract was measured for all birds as described previously^23^. Gut contents (10 g) were aseptically collected from the dissected jejunum and ileum of each bird and placed in 90 mL sterilised physiological saline (1:10 dilution; Terumo Corporation, Tokyo, Japan), and the pH was determined using a pH meter (HORIBA Ltd., Kyoto, Japan).

### Microbial enumeration

The microbial counts of jejunum and ileum were obtained as previously described^24^. Approximately 1 g of jejunal and ileal digesta was obtained from all birds and serially diluted 10-fold (from 10^−1^ to 10^−7^) with sterile physiological saline solution (0.9% NaCl) and subsequently homogenised for 3 min by using an ultra-turrax. Dilutions were then plated onto selective agar medium for enumeration of the target bacterial groups. *Escherichia coli* were grown on MacConkey agar (Beijing Aoboxing Bio-tech Co., Ltd., Beijing, China). Lactobacilli were cultivated using de Man–Rogosa–Sharpe agar (Oxoid Ltd., Hampshire, UK). *Lactobacillus* plates were incubated anaerobically, whereas *E. coli* plates were incubated aerobically at 37 °C for 24 h. Bacteria were enumerated by visual counting of colonies by using the best replicate set from dilutions that resulted in 30 to 300 colonies per plate. The microbial enumerations of jejunum and ileum were expressed as base-10 logarithm colony-forming units per gram of jejunum and ileum digesta.

### Serum α-tocopherol concentration

For the analysis of serum α-tocopherol concentrations, blood samples of all birds were collected from the wing vein and allowed to clot for 30 min. Blood clots were centrifuged at 3,000 × *g* for 15 min at 4 °C; the top yellow serum layer was pipetted into two 1-mL conical tubes and held at −80 °C. The α-tocopherol level in the serum was determined using high-performance liquid chromatography^25^.

### Reactive oxygen metabolites and biological antioxidant potential

The reactive oxygen metabolite-derived compound (d-ROM) test provides a measure of the whole oxidant capacity of plasma against *N*,*N*-diethylparaphenylendiamine in acidic buffer. Such oxidant capacity is mainly attributed to hydroperoxides, with the contribution of other minor oxidant factors. The biological antioxidant potential (BAP) test evaluates the capacity of the plasma sample to reduce ferric ions to ferrous ions. BAP varies primarily as a function of the titres of the major oxidative barriers in the plasma (vitamin C, vitamin E, uric acid, bilirubin, etc.). For the analysis d-ROMs and BAP, blood samples of all birds were collected from the wing vein in EDTA-containing blood collection tubes and centrifuged (1,000 × *g* for 15 min). The plasma supernatants were stored at −80 °C until assayed. The d-ROMs and BAP were determined using commercial kits (Diacron, Grosseto, Italy) and FRAS4 (H & D, Parma, Italy), respectively^26^.

### Semen traits

The semen from all birds from each group was collected using the massage method as per the procedure of Burrows and Quinn^27^. The spermatozoa density or sperm count in the semen was estimated using a colorimetric method^28^. The live and dead spermatozoa were differentiated by staining with eosin and nigrosine by using the method described by Swanson and Bearden^29^.

### Statistical analysis

Mann–Whitney *U* tests were performed using EZR software (Saitama Medical Center, Jichi Medical University); EZR is a graphical user interface for R (The R Foundation for Statistical Computing, version 2.13.0). The significance level was set at *p* < 0.05.

## Results

The villus height, crypt depth, and villus height:crypt depth ratio from each group are shown in Table 2. The crypt depth of jejunum and ileum was not markedly different between groups 1 and 2. In contrast, the villus height and villus height:crypt depth ratio of the jejunum and ileum were significantly higher in group 2 than in group 1. The pH of the digestive tract contents from the jejunum and ileum was significantly lower in group 2 than in group 1 (Table 2).

**Table 2:** Small intestinal morphology and pH of digestive tract contents (mean ± SD).

		group 1 (control)	group 2 (probiotics)
Jejunum	Villus height (µm)	1144.9 ± 144.7^a^	1217.0 ± 134.4^b^
	Crypt depth (µm)	300.7 ± 71.2	294.2 ± 72.3
	Villus height to crypt depth ratio	4.06 ± 1.27^a^	4.39 ± 1.19^b^
	pH	6.65 ± 0.18^a^	6.45 ± 0.14^b^
Ileum	Villus height (µm)	490.1 ± 106.8^a^	525.0 ± 44.4^b^
	Crypt depth (µm)	150.6 ± 14.7	148.9 ± 15.6
	Villus height to crypt depth ratio	3.28 ± 0.75^a^	3.56 ± 0.46^b^
	pH	6.84 ± 0.22^a^	6.70 ± 0.18^b^

^a,b^Different letters within rows indicate differences between treatment groups (*p* < 0.05).

The viable counts of *Lactobacillus* were significantly higher in group 2 than in group 1, whereas those of *E. coli* were significantly lower in group 2 than in group 1 (*p* < 0.05; Table 3). The serum α-tocopherol concentration, BAP, and sperm density, and proportion of live sperms were significantly higher in group 2 than in group 1 (Tables 4,5). However, the d-ROM levels were not markedly different between groups 1 and 2 (Table 4).

**Table 3:** Microbial enumeration (log cfu/g of wet digesta; mean ± SD).

		group 1 (control)	group 2 (probiotics)
Jejunum	Lactobacillus	7.50 ± 0.59^a^	7.82 ± 0.59^b^
	*Escherichia coli*	6.84 ± 0.56^a^	6.42 ± 0.37^b^
Ileum	Lactobacillus	7.39 ± 0.53^a^	7.68 ± 0.30^b^
	*Escherichia coli*	6.59 ± 0.27^a^	6.49 ± 0.30^b^

^a,b^Different letters within rows indicate differences between treatment groups (*p* < 0.05).

**Table 4:** α-Tocopherol concentration, reactive oxygen metabolites, and biological antioxidant potential (mean ± SD).

	group 1 (control)	group 2 (probiotics)
α-tocopherol concentration in serum (µg/mL)	2.603 ± 0.774^a^	2.990 ± 0.750^b^
Reactive oxygen metabolites in plasma (Carratelli units)	29.9 ± 1.20	30.1 ± 1.13
Biological antioxidant potential in plasma (µmol/L)	2683.4 ± 201.9^a^	2896.8 ± 333.9^b^

^a,b^Different letters within rows indicate differences between treatment groups (*p* < 0.05).

**Table 5:** Semen traits (mean ± SD).

	group 1 (control)	group 2 (probiotics)
Sperm count (million/mL)	22.8 ± 2.55^a^	26.5 ± 2.90^b^
Live sperm (%)	94.1 ± 2.63^a^	95.2 ± 1.06^b^

^a,b^Different letters within rows indicate differences between treatment groups (*p* < 0.05).

## Discussion

The major microbes used as probiotics in poultry production include *Lactobacillus*, *Saccharomyces*, *Streptococcus*, *Aspergillus* spp., and *Bacillus*^30^; their success in providing beneficial effects to the host depends on their ability to tolerate heat, osmotic stress, and oxygen stressors during processing and storage^31^. *B. amyloliquefaciens* are spore-forming bacteria having resistance to high temperature and harsh storage conditions and are generally regarded as safe for use as probiotics in poultry production^32–35^. Several studies have shown favourable results with broilers by using *B. amyloliquefaciens*^32–35^. In the present study, *B. amyloliquefaciens* TOA5001 was mixed with a carrier (rice bran) such that the addition of 2 g·kg^−1^ of diet would yield 10^8^ colony-forming units per kg diet. Probiotics are known to be efficacious in animals at the daily intake level of 10^7^–10^9^ microorganisms^36–38^.

In this study, dietary supplementation with *B. amyloliquefaciens* TOA5001 did not show any adverse effects on male broiler breeders. The structure and integrity of the intestinal epithelium are important factors contributing to gut health and subsequent digestive capacity. Villus height is generally recognised as a good indicator of the function and activation of intestinal villi^39^. Better villus height and villus height to crypt depth ratio suggest an improvement in nutrient digestibility and absorption capacity of the small intestine^40^. The present study showed that *B. amyloliquefaciens* TOA5001 improved the gut structure and resulted in a greater absorption surface, as indicated by the improved villus height and villus height to crypt depth ratio. The effects of probiotics on gut structure and integrity have also been reported in the literature. Jayaraman *et al*.^41^. and Xinjian *et al*.^32^, respectively, reported that the inclusion of *B. subtilis* and *B. amyloliquefaciens* in broiler diets led to better villus height and villus height to crypt depth ratio. These two factors have been shown to be related to the epithelial cell turnover^42^. Inflammatory responses induced by pathogens or their toxins might cause the rapid epithelial cell turnover^43^. Thus, the suppression of pathogenic bacteria by *B. amyloliquefaciens* TOA5001 might have resulted in the better villus height and villus height to crypt depth ratio.

In the gastrointestinal tract, numerous microorganisms co-exist and constitute a symbiotic ecosystem in equilibrium^44^. Various studies have shown that probiotics can positively modulate the composition of the intestinal microflora of chickens via the stimulation of potentially beneficial bacterial populations and/or the reduction of potentially pathogenic bacteria^45^. In the present study, jejunum and ileum samples of birds fed a diet containing *B. amyloliquefaciens* TOA5001 had lower pH, higher *Lactobacillus* concentration, and reduced *E. coli* counts. The *B. amyloliquefaciens* TOA5001-mediated reduction in intestinal pH might be favourable for the colonisation of lactobacilli and the suppression of *E. coli*^46^.

Mitsuoka^47^ indicated that irregular intestinal microflora can cause malabsorption of vitamins. In mouse, rotavirus infection was shown to cause acute diarrhoea and vitamin deficiency^48^. In the present study, serum vitamin E (α-tocopherol) concentrations were significantly higher in group 2 than in group 1. Probiotics have been shown to increase serum vitamin E concentration in cattle by improving the intestinal environment^49^. Thus, the probiotics containing *B. amyloliquefaciens* TOA5001 might improve the digestive health of male broiler breeders.

Reactive oxygen metabolites are produced as a by-product of oxidative metabolism or exposure to oxidants in food or the environment. Oxidant exposure leads to the production of toxic reactive oxygen species such as free radicals, which in turn modify biological macromolecules. Vitamin E is known as an excellent biological chain-breaking antioxidant that protects cells and tissues from lipid peroxidation induced by free radicals^50,51^. It also increased plasma BAP in sheep exposed to heat stress^52^. However, few studies have linked BAP and probiotic supplements in chickens. In the present study, although d-ROM was not markedly different between groups 1 and 2, BAP was significantly higher in group 2 than in group 1. These data indicate that male broiler breeders in groups 1 and 2 were under the same oxidative stress conditions, but probiotic supplements increased the antioxidative activity of the male broiler breeders. *B*. *amyloliquefaciens* TOA5001 supplementation was suggested to increase serum vitamin E concentrations and improve the antioxidative activity of male broiler breeders by increasing antioxidant absorption in the intestine.

In the present study, the sperm density and proportion of live sperms were significantly higher in group 2 than in group 1. The results of sperm density are in agreement with those of a previous study in which broiler breeders were fed diets supplemented with yeast culture^53^. The results of the proportion of live sperms was in agreement with that of a previous study in which broiler breeders after zinc-induced moulting were fed diets supplemented with probiotics^54^. The observed improvement in sperm concentration in the *B. amyloliquefaciens* TOA5001-fed males might have been due to the enhanced availability of nutrients facilitated by more efficient nutrient absorption by the gastrointestinal tract. Furthermore, several studies have indicated higher antioxidant activity in chickens fed probiotic-supplemented diets^23,55^, and *B. amyloliquefaciens* TOA5001 improved the antioxidative activity of male broiler breeders in this study. Thus, the relationship between the improvement in the activity of antioxidants such as glutathione peroxidase (GSH-Px) and superoxide dismutase in *B. amyloliquefaciens* TOA5001-fed birds and spermatozoa production and maturation needs to be considered. High levels of GSH-Px are found in the testes, and they act as powerful antioxidants in developing spermatids and spermatozoa^56^. Spermatozoa are adversely affected by high concentrations of peroxides in the testes, semen, and uterovaginal sperm host glands^57,58^. In organs such as testes that have high metabolic rates, levels of antioxidants required to ensure the survival of spermatozoa in aerobic environments are high. Thus, the high density of spermatozoa and high proportion of live sperms recorded in *B. amyloliquefaciens* TOA5001-fed males in this study might be attributed to the influence on the antioxidant activity.

## Conclusions

Our results indicate that probiotics containing *B. amyloliquefaciens* TOA5001 improve intestinal morphology, intestinal microflora, oxidative activity (biological antioxidant potential), and semen quality (sperm count and liver sperm) of male broiler breeders. Therefore, their use would be advantageous to the poultry industry.
